# Low profile posterior lumbar-sacral interbody fusion for lumbosacral degenerative diseases: a technical note

**DOI:** 10.1186/s12891-023-06993-8

**Published:** 2023-11-14

**Authors:** Aixing Pan, Fengqi Cheng, Zihao Ding, Li Guan, Wenguan Xie, Yong Hai, Yuzeng Liu

**Affiliations:** 1 Department of Orthopedic Surgery, Beijing Chao-Yang Hospital, Capital Medical University, No. 8 Gongtinan Road, Beijing, 100020 China; 2 Department of Orthopedics, Dongguan Eighth People’s Hospital, Dongguan, Guangdong Province 523320 China

**Keywords:** Low-profile, Posterior lumbosacral interbody fusion, Minimally invasive, Surgical technique

## Abstract

**Background:**

The purpose of this study was to report our surgical experience in patients with lumbosacral degenerative diseases who underwent posterior decompression and interbody fusion fixed with cortical bone trajectory screw and sacral alar screw, which is known as low-profile posterior lumbosacral interbody fusion (LP-PLSIF).

**Methods:**

Patients with lumbosacral degenerative disease who underwent LP-PLSIF and traditional PLSIF (control group) internally fixed with pedicle screws were included retrospectively. Patients’ demographic data, operative parameters, and perioperative complications were recorded and analyzed.

**Results:**

A total of 18 patients were enrolled in this study, which included 9 patients (5 male and 4 female) who underwent LP-PLSIF, and 9 patients (4 male and 5 female) who underwent traditional PLSIF. There wasn’t a significant difference in the average age between the two groups, 56.78 ± 10.92 years in the LP-PLSIF group and 60.22 ± 8.21 years in the PLSIF group (*p* = 0.460). The bone mineral density (BMD) of the two groups of patients were -2.00 ± 0.26 T and -2.13 ± 0.19 T, respectively (*P* = 0.239). The mean postoperative follow-up time was 12.7 months (range, 12–14 months). The mean operation time was 142.78 ± 11.21 min and 156.11 ± 13.41 min in the LP-PLSIF group and PLSIF group respectively (*P* < 0.05). The average blood loss was 137.78 ± 37.09 ml in the LP-PLSIF group, and 150.00 ± 27.84 ml in the PLSIF group (*P* = 0.441). The average postoperative drainage was 85.56 ± 37.45 ml and 122.22 ± 22.24 ml in the LP-PLSIF group and control group respectively (*P* < 0.05). Patients in the LP-PLSIF group had shorter incision length compared with the control group, 61.44 ± 10.56 mm vs. 74.56 ± 10.22 mm (*P* < 0.05). The average length of hospitalization of 11.33 ± 2.92 days in the LP-PLSIF group, and 11.11 ± 1.62 days in the PLSIF group (*p* = 0.844). All patients had significant improvement in VAS pain score, ODI, and JOA evaluation. However, patients in the LP-PLSIF group had better improvement in terms of VAS back pain and ODI in the short term after the operation. There were no neurological complications or wound infection. The fusion rate at the last follow-up was 100% (9 of 9) in the LP-PLSIF group, and 88.89% (8 of 9) in the control group based on CT scans. 1 patient in the control group had asymptomatic sacral pedicle screw loosening.

**Conclusions:**

LP-PLSIF is a safe and effective surgical technique for patients with lumbosacral degenerative disease, which has the potential strength of less invasive and better clinical improvement.

## Background

As the aging of the population, the annual prevalence of osteoporosis or osteopenia with lumbar degenerative diseases is increasing, resulting in heavy health and economic burden both on families and society [1, 2]. The lumbosacral region has the highest incidence of lumbar degenerative diseases due to greater stress and mobility. However, the highest incidence of internal fixation failure occurred in the lumbosacral region after lumbar internal fixation, especially in the osteoporotic population [3]. Therefore, improving the internal fixation and reducing the mechanical complications has consistently been a challenge in spinal fusion surgery.

The most commonly used internal fixation method for posterior lumbar spine fusion is the pedicle screw (PS) fixation technique [4]. PS penetrates the three columns of the vertebral body to achieve three-column fixation, which is characterized by high stability, better orthopedic effect, and high fusion rate [5]. However, in the treatment of elderly patients with osteoporosis, internal fixation with the PS is prone to screw loosening, internal fixation failure, and high revision rate [6, 7]. In addition, the PS technique is associated with extensive soft tissue and paravertebral muscle exposure, which increases surgical trauma and delays recovery after the operation.

**Fig. 1:**
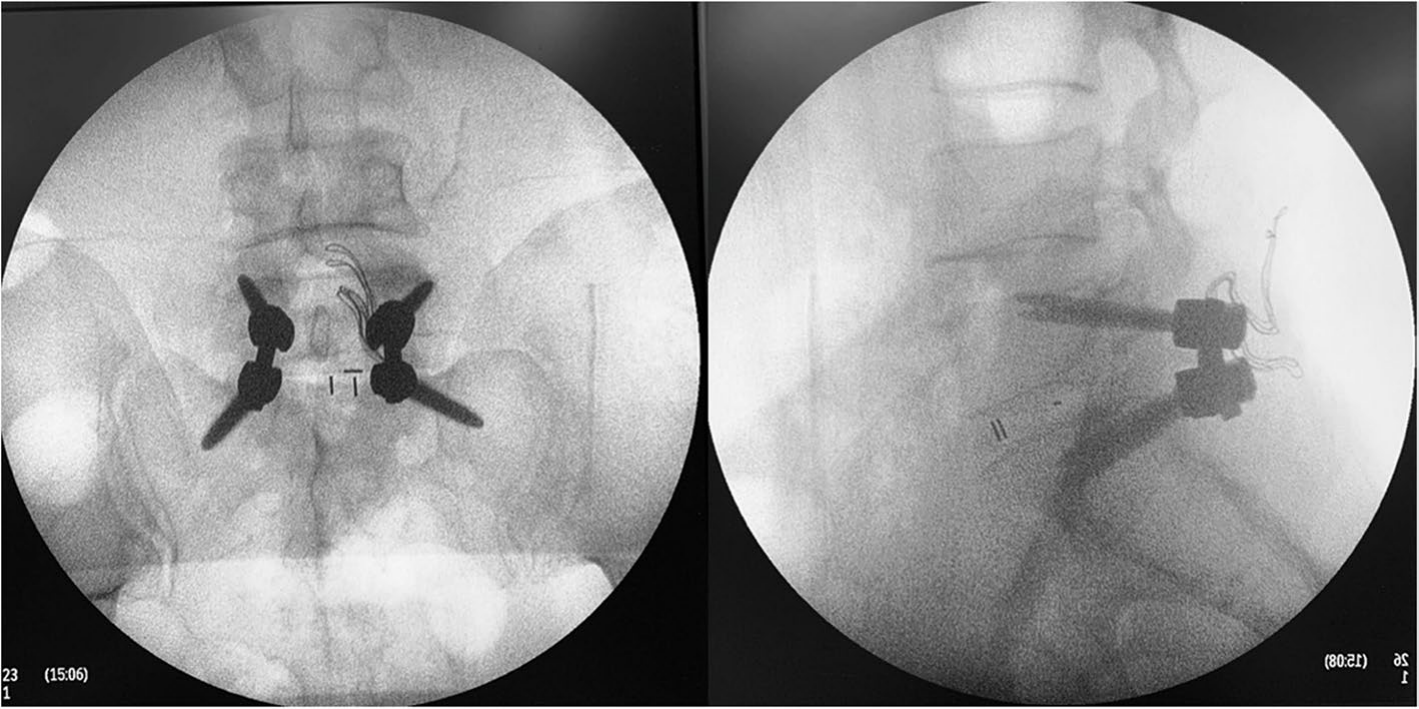
Intraoperative radiographs of L5/S1 LP-PLSIF surgery. L5 is fixed with CBTS and S1 is fixed with SAS

To improve internal fixation and reduce surgical trauma, we developed low-profile posterior lumbosacral interbody fusion (LP-PLSIF) technique (Fig. 1), which combines lumbar cortical bone trajectory screw (CBTS) and sacral alar screw (SAS) internal fixation techniques in lumbosacral fusion surgery [8]. Previous research demonstrated that the CBTS technique had a significantly lower incidence of postoperative complications and revision rate along with better clinical improvement in posterior lumbar interbody fusion surgery for osteoporotic patients than the PS fixation technique [3, 9, 10]. As the SAS screw has a longer trajectory and a larger abduction angle, it can significantly improve the anti-extraction force and maintain the good stability of internal fixation. In addition, the SAS screw could get a stronger anchor through additional purchase in the sacral cortical bone [11]. In this study, we described our initial experience of LP-PLSIF surgery in lumbosacral degenerative patients. Surgical data and postoperative outcomes were recorded and analyzed.

## Materials and methods

### Surgery technique

#### Inclusion and exclusion criteria

This study was approved by the appropriate ethics review board of the authors’ hospital. The study was carried out based on the data of 18 consecutive patients who underwent lumbar fusion surgery for degenerative spine disease in our institution between 2020 and 2022. Nine of the patients underwent LP-PLSIF procedure and the other nine patients underwent traditional open PLSIF procedure as a control group. The included patients were all associated with decreased bone mineral density, and informed consent from patients was obtained. The inclusion criteria were listed below: (1) diagnosed as lumbar disc herniation (LDH) or lumbar spinal stenosis (LSS); (2) diagnosed as decreased bone mineral density; (3) underwent posterior L5/S1 interbody fusion internal fixation surgery; (4) at least 12 months of postoperative follow-up. The exclusion criteria were listed below: (1) lumbar spondylolisthesis or scoliosis; (2) spinal metastases; (3) spinal fracture; (4) lumbar fusion surgery history; (5) patients with psychiatric diseases or voluntary withdrawal. After surgery, all cases were given the same postoperative care and rehabilitation program.

### Illustrative case

**Fig. 2:**
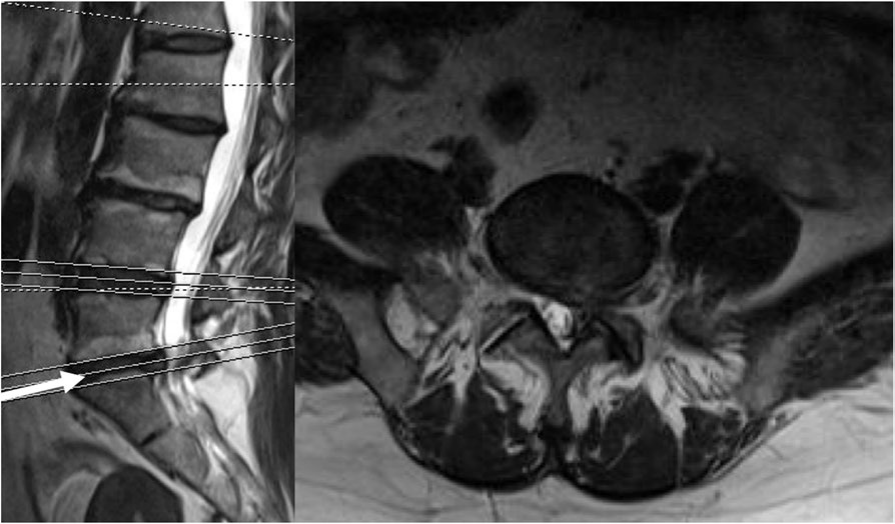
Preoperative MRI showed L5/S1 LSS caused by disc herniation and hypertrophy of the facet joint on the left side

**Fig. 3:**
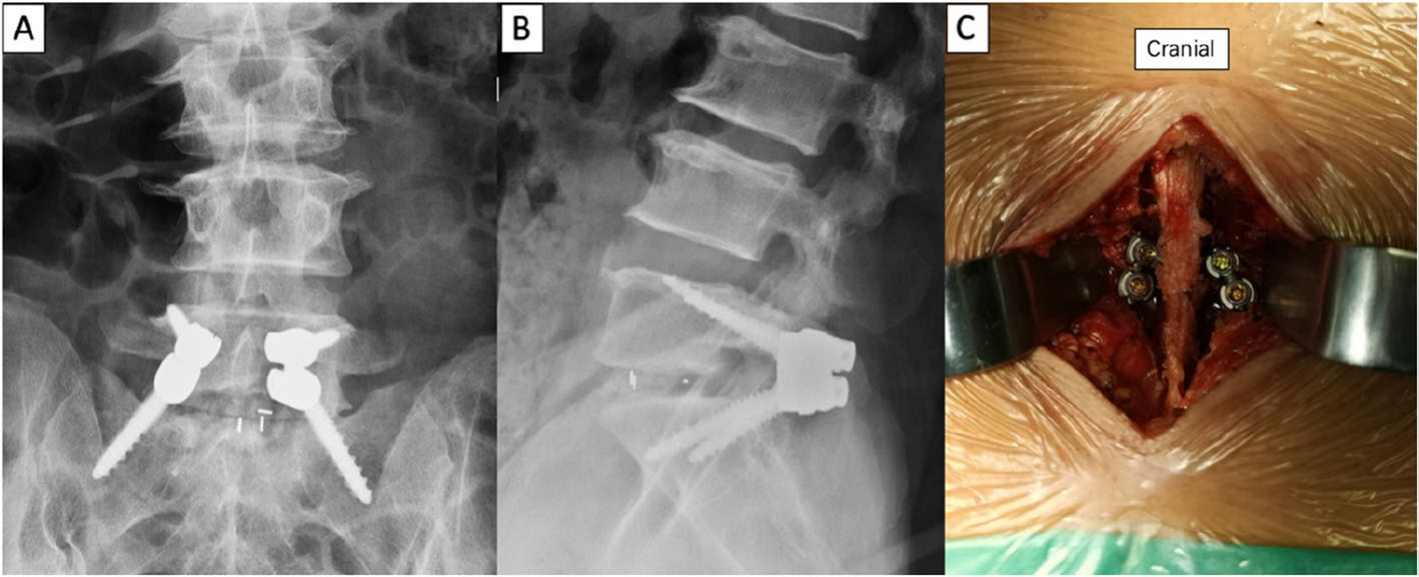
**A** LP-PLSIF postoperative coronal lumbar spine X-ray. **B** Postoperative sagittal lumbar spine X-ray. **C** LP-PLSIF intraoperative picture

It’s a 54-year-old female patient who was diagnosed with lumbar spinal stenosis (LSS) and decreased bone mineral density (BMD = -1.56). She had a surgical history of endoscopic lumbar discectomy at L5/S1 1 year ago. Preoperative lumbar spine MRI showed the L5/S1 disc herniation and hypertrophy of the facet joint on the left side (Fig. 2). She responded poorly to the conservative treatment and underwent posterior L5/S1 decompression and interbody fusion surgery with the LP-PLSIF technique (Fig. 3).

#### Surgical planning

The surgical plan was made based on the preoperative evaluation of imaging findings and patient symptoms. Posterior lumbar decompression interbody fusion was performed via a posterior midline incision. The autograft bone was obtained locally during laminectomy and facetectomy, and a polyether ether ketone interbody cage with a built-in autologous bone was inserted between the intervertebral space after bone grafting. Internal fixation was performed under fluoroscopic guidance.

#### Positioning and anesthesia

The patient was placed in a prone position on an operating table using thoracic and iliac pads to restore the lordosis of the lumbar spine while avoiding pressure on the peritoneal cavity. Additionally, the height and orientation of the surgical table were adjusted to facilitate intraoperative C-arm fluoroscopy. The intravenous access was prepared along with the necessary monitoring instruments. First, pressurized pure oxygen inhalation was performed using a mask for 3–5 min, followed by insertion to the trachea through the voice portal. Anesthetic drugs were administered to the patient through a combination of tracheal intubation and intravenous channels, which produced temporary central nervous system depression in the patient.

#### Incision and exposure

An incision of approximately 5 cm was made in the posterior midline. After the incision, the soft tissues and muscles were dissected and separated layer-by-layer to expose the L5/S1 lamina and facet joints.

#### CBTS fixation

**Fig. 4:**
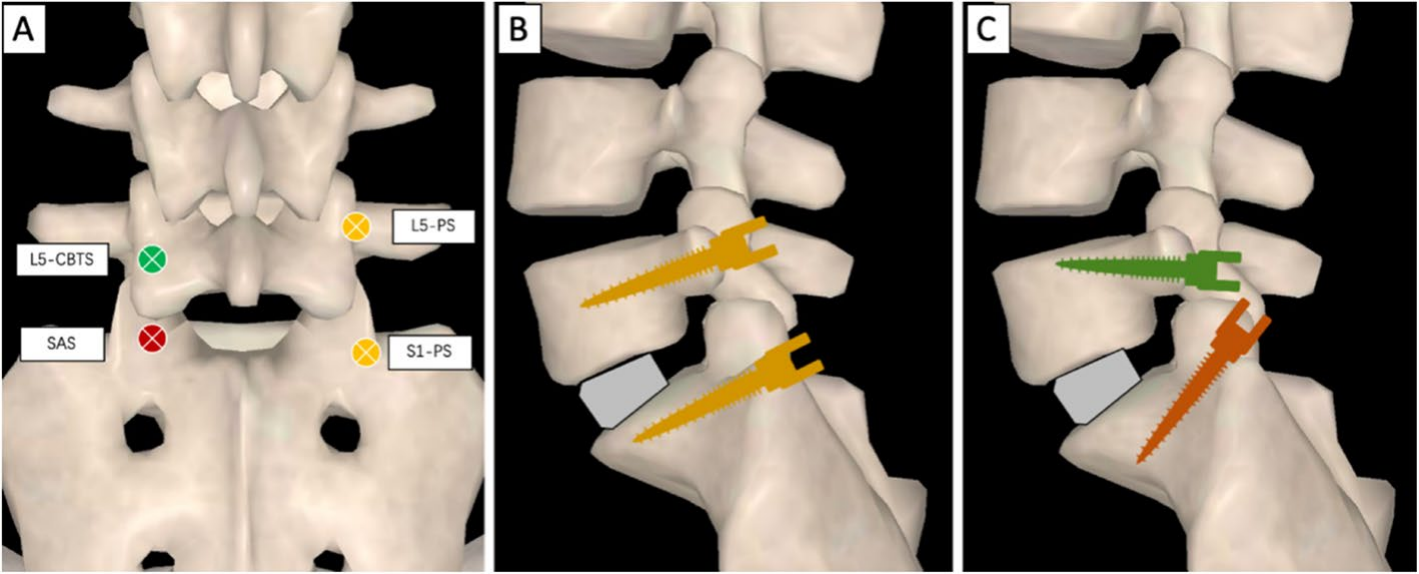
The insertion point of PS, CBTS, and SAS (**A**). The different screw trajectories of L5/S1 traditional PLSIF (**B**) and LP-PLSIF (**C**)

The starting point was located approximately 2–3 mm below the edge of the L5 transverse process and 2–3 mm inside the edge of the L5 isthmus (Fig. 4). An insertion hole was then made by drill in a medial-to-lateral and caudal-to-cranial direction under C-arm guidance. Generally, the screw trajectory was inclined 30–40° to the caudal and 15–20° to the medial. A screw tap was then used to make the screw trajectory. CBTS were inserted after decompression and interbody fusion.

#### Wound closure and drainage

A drainage tube was placed beneath the paraspinal muscles. The incised soft tissue and skin were carefully closed layer-by-layer.

## Results

### Patient characteristics

During March 2020 and March 2022, 18 patients were enrolled in this study, which included 9 male patients and 9 female patients. Nine patients (7 lumbar spinal stenosis and 2 lumbar disc herniation) were treated with LP-PLSIF, and 9 patients (7 lumbar spinal stenosis and 2 lumbar disc herniation) underwent traditional PLSIF as a control group. The mean age of the participants was 56.78 ± 10.92 years old (range, 41–75 years), and 60.22 ± 8.21 years old (range, 47–72 years) in the LP-PLSIF and PLSIF groups respectively. Surgeries were performed by one experienced spine surgeon. Perioperative clinical presentations, operative variables, and surgical complications were recorded.

### Surgical parameters

**Fig. 5:**
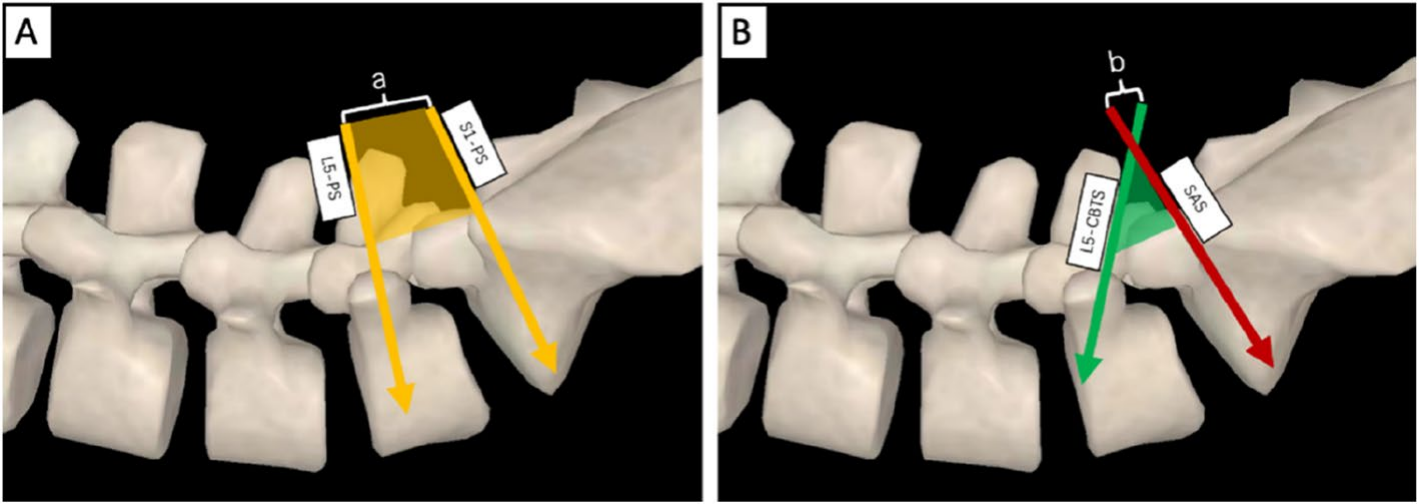
**A** The incision length (a) and the surgical exposure area (yellow shadow) of traditional PLSIF surgery. **B** The incision length (b) and the surgical exposure area (green shadow) of LP-PLSIF surgery

**Fig. 6:**
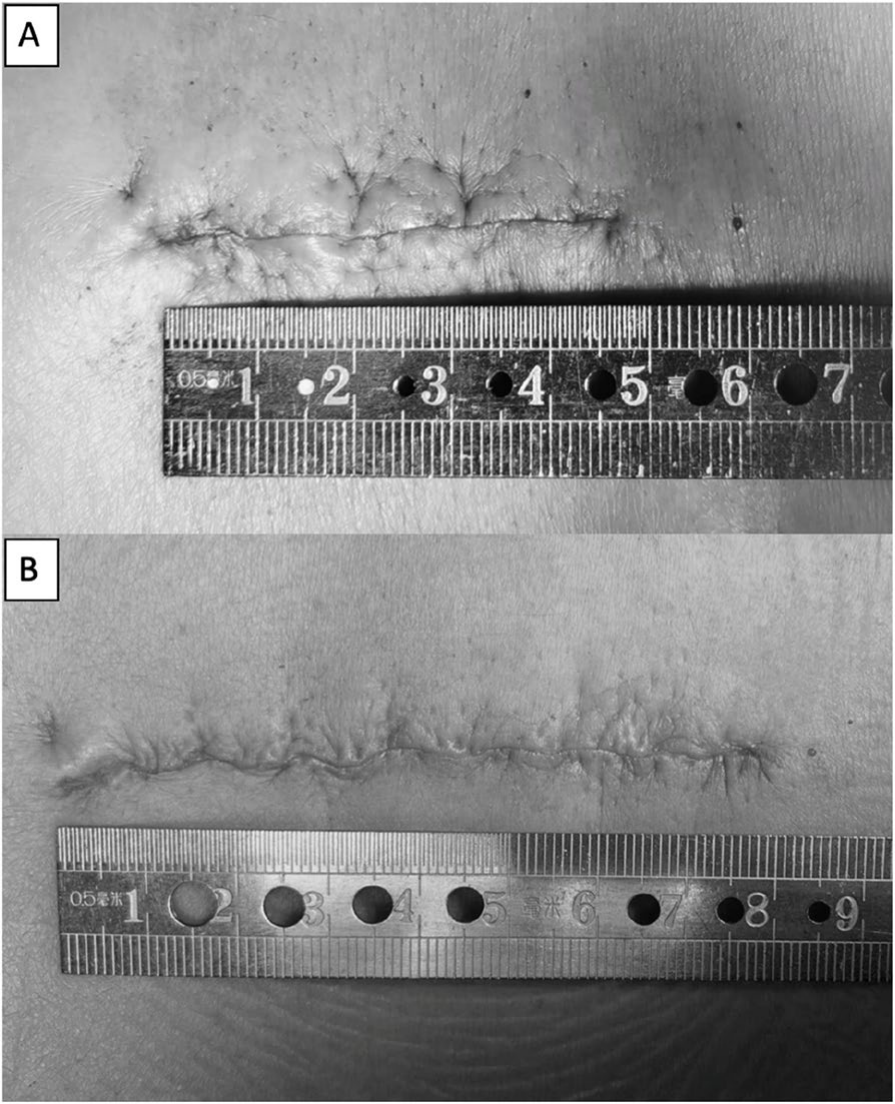
The incision length of LP-PLSIF surgery (**A**) and traditional PLSIF surgery (**B**)

The mean operative time was 142.78 ± 11.21 min (range, 120–160 min) in the LP-PLSIF group and 156.11 ± 13.41 min (range, 135- 180 min) in the PLSIF group (*P* < 0.05). The average blood loss was 137.78 ± 37.09 ml (range, 80–180 ml) in the LP-PLSIF group, and 150.00 ± 27.84 ml (range, 100–200 ml) in the PLSIF group (*P* = 0.441). No patient required a blood transfusion. The average postoperative drainage was 85.56 ± 37.45 ml (range, 40–130 ml) and 122.22 ± 22.24 ml (range, 80–150 ml) respectively in the LP-PLSIF group and control group (*P* < 0.05). The average incision length was 61.44 ± 10.56 mm (range, 52-84 mm) and 74.56 ± 10.22 mm (range, 65-96 mm) respectively in the LP-PLSIF group and control group (*P* < 0.05) (Figs. 5 and 6). The average length of hospitalization was 11.33 ± 2.92 days in the LP-PLSIF group, and 11.11 ± 1.62 days in the PLSIF group (*p* = 0.844) (Tables 1 and 2). No patient had nerve injury, dural tear, or wound infection.

**Table 1:** Patient information and surgical parameters of LP-PLSIF group

Case No.	Sex/Age	Diagnosis	Operation time (min)	Intraoperative blood loss (ml)	Postoperative drainage (ml)	Incision length (mm)	Hospital stay (day)	BMD
1	M/54	LSS	145	150	130	55	9	-1.56T
2	F/51	LSS	140	145	120	63	12	-2.27T
3	F/57	LSS	150	180	100	56	13	-2.43T
4	M/70	LSS	145	120	110	52	10	-1.89T
5	M/61	LSS	160	175	60	84	18	-1.74T
6	M/57	LDH	120	80	40	57	9	-2.02T
7	F/45	LSS	135	80	40	62	10	-2.09T
8	M/75	LSS	150	160	120	72	12	-1.95T
9	F/41	LDH	140	150	50	52	9	-2.03T
Mean value ± SD	56.78 ± 10.92		142.78 ± 11.21	137.78 ± 37.09	85.56 ± 37.45	61.44 ± 10.56	11.33 ± 2.92	-2.00 ± 0.26

**Table 2:** Patient information and surgical parameters of PLSIF group

Case No.	Sex/Age	Diagnosis	Operation time (min)	Intraoperative blood loss (ml)	Postoperative drainage (ml)	Incision length (mm)		Hospital stay (day)	BMD
1	F/56	LSS	155	150	130	67		11	-2.41T
2	M/62	LDH	150	150	130	76		10	-2.07T
3	F/57	LSS	160	200	150	69		13	-2.47T
4	M/68	LSS	145	140	120	65		10	-1.98T
5	F/62	LSS	180	180	150	96		12	-2.12T
6	M/67	LSS	135	140	120	70		11	-1.94T
7	F/51	LSS	150	100	80	74		10	-2.12T
8	M/72	LDH	170	140	120	86		14	-2.08T
9	F/47	LSS	160	150	100	68		9	-1.97T
Mean value ± SD	60.22 ± 8.21		156.11 ± 13.41	150.00 ± 27.84	122.22 ± 22.24		74.56 ± 10.22	11.11 ± 1.62	-2.13 ± 0.19

### Follow-up parameters

Patients were followed up at least 12 months after surgery. The mean follow-up time was 12.7 months (range, 12–14 months). No screw loosening or internal fixation fracture was observed based on computed tomography (CT) imaging during the follow-up in the LP-PLSIF group. One patient had radiographic sacral pedicle screw loosening without symptoms in the control group. The fusion rate at a mean 12-month follow-up was 100% (9 of 9) in the LP-PLSIF group and 88.89% (8 of 9) in the PLSIF group on CT scans.

Twelve-month improvements in back pain and lower limb pain were evaluated using a visual analog scale (VAS). In the LP-PLSIF group, the mean VAS (leg pain) score was 7.00 ± 0.87 preoperatively, dropping to 2.78 ± 0.67 in the first month follow-up, and 1.33 ± 0.50 in the final follow-up. In the PLSIF group, the VAS (leg pain) score was 6.78 ± 0.67 preoperatively, dropped to 2.89 ± 0.60 at the first month follow-up, and 1.44 ± 0.53 at the last follow-up, with no significant difference between two groups. In the LP-PLSIF group, the average VAS (back pain) score of was 6.22 ± 0.83 preoperatively, decreased to 2.22 ± 0.67 in the first month follow-up, and 0.89 ± 0.33 in the final follow-up in the LP-PLSIF group. In the PLSIF group, the VAS (back pain) score was 6.44 ± 0.53 preoperatively, decreased to 3.00 ± 0.71 at the first month follow-up, and 1.56 ± 0.53 at the last follow-up. In the LP-PLSIF group, the mean Oswestry Disability Index (ODI) was significantly improved from 63.56 ± 5.55% to 19.56 ± 2.83% at the final follow-up (*p* < 0.05). In the PLSIF group, ODI was also significantly improved from 64.56 ± 5.03% to 22.33 ± 2.35% (*p* < 0.05). The average Japanese Orthopedic Association (JOA) score was improved from 12.67 ± 2.35 to 2.67 ± 2.35, and 11.89 ± 2.26 to 22.00 ± 1.50 in the LP-PLSIF group and PLSIF group respectively (*p* = 0.483). Patients in the LP-PLSIF group had better improvement in terms of postoperative VAS back pain and ODI (*p* < 0.05) (Table3).

**Table 3:** Clinical outcome evaluation in the LP-PLSIF and PLSIF groups

	LP-PLSIF group	PLSIF group	*P*
**VAS (leg pain)**			
Pre-operation	7.00 ± 0.87	6.78 ± 0.67	0.550
Post-operation	2.78 ± 0.67	2.89 ± 0.60	0.715
12-month follow-up	1.33 ± 0.50	1.44 ± 0.53	0.653
P#	0	0	/
**VAS (back pain)**			
Pre-operation	6.22 ± 0.83	6.44 ± 0.53	0.509
Post-operation	2.22 ± 0.67	3.00 ± 0.71	0.029*
12-month follow-up	0.89 ± 0.33	1.56 ± 0.53	0.007*
P#	0	0	**/**
**Oswestry Disability Index score (ODI)**			
Pre-operation	63.56 ± 5.55	64.56 ± 5.03	0.694
12-month follow-up	19.56 ± 2.83	22.33 ± 2.35	0.038*
P#	0	0	/
**Japanese Orthopaedic Association score (JOA)**			
Pre-operation	12.67 ± 2.35	11.89 ± 2.26	0.484
12-month follow-up	22.67 ± 2.35	22.00 ± 1.50	0.483
P#	0	0	/

*Statistically significant

*Abbreviations*: *VAS* Visual Analogue Scale, *ODI* Oswestry Disability Index, *JOA* Japanese Orthopaedic Association Score

## Discussion

Optimal internal fixation for posterior lumbar fusion surgery remains controversial because of the increased incidence of degenerative lumbar spine disease along with decreased bone mineral density [12, 13]. Currently, PS internal fixation is broadly used in clinical practice and has been an effective treatment for posterior lumbar spine fusion [14]. However, especially in the treatment of patients with osteoporosis, high surgery-related complications and revision surgery rates have led to heavy financial and emotional burdens and unsatisfactory postoperative outcomes in some patients with lumbar degenerative diseases [9, 12]. Because most of the trajectory of the PS passes through the cancellous bone, internal fixation with PSs is prone to screw loosening during the treatment of elderly patients with osteoporosis, resulting in internal fixation failure and a high rate of surgical rework [10]. In addition, PS internal fixation is always associated with more invasive, such as extensive soft-tissue and paravertebral muscle exposure and superior facet-joint violation, which leads to failure in posterior stabilization and delayed recovery after operation [15]. Thus, obtaining a solid fusion and minimizing the invasiveness to soft tissues has consistently been pursued in lumbar fusion surgery.

Therefore, CBTS alone or in combination with PS for internal fixation has become increasingly popular for treating patients with osteoporosis [16]. In our experimental and clinical results, poor surgical outcomes related to PS internal fixation were optimized and improved significantly using CBTS internal fixation [9]. CBTSs have a unique screw placement trajectory that follows a caudocephalad path in the sagittal plane and a mediolateral path in the transverse plane [17]. This unique trajectory can be used in patients with osteoporosis or osteopenia to minimize the risk of internal fixation loosening. Biomechanical studies have shown that CBTSs have a greater mean pullout strength and that the uniaxial pullout resistance of CBTSs is 30% higher than that of PSs [7, 18]. In addition, the stiffness in cephalocaudal or mediolateral loading and resistance to flexion/extension were significantly stronger when using CBT rescue screws than when using traditional PSs [19]. Moreover, CBTSs were efficient in avoiding extended damage to the soft tissue and superior facet joint and minimizing ischemic necrosis and denervation of the posterior muscles, which was effective in enhancing fusion rates and maintaining stability in patients with osteoporosis [20, 21]. Therefore, the perioperative parameters of CBTSs were superior to those of traditional PSs, including blood loss, skin incision, and surgical time.

The increased incidence of lumbosacral internal fixation failure is a major concern in lumbosacral spinal fusion surgery [22]. Achieving solid lumbosacral fusion is a big challenge for lumbosacral fusion due to the poor bone quality of the sacrum, complex anatomy, and substantial biomechanical shear forces of the lumbosacral junction [23]. The biomechanical characteristics induce a risk of pseudarthrosis and adjacent segment degeneration in this region, which may lead to failure of lumbosacral internal fixation [24, 25]. Since the introduction of the Galveston iliac fixation instrument in 1982, emerging sacroiliac fixation techniques, such as sacral alar screw(SAS), have been recognized as reliable fixation methods for lumbosacral internal fixation surgery [26]. Previous experimental and clinical results have demonstrated that SAS can not only avoid sacroiliac joint violation but also enhance stability [11, 26]. Therefore, we proposed the LP-PLSIF technique which combined CBTS and SAS techniques, aiming to reduce the surgical invasiveness and improve the internal fixation stability [25].

Compared to the conventional PS technique, the LP-PLSIF technique requires less surgical time (142.78 ± 11.21 min, *P* < 0.001), less incision length (61.44 ± 10.56 mm, *P* < 0.001), and less blood loss (137.78 ± 37.09 mL, *P* = 0.004) while achieving better results for pain relief and functional recovery. Possible complications related to the PS technique, such as screw loosening and internal fixation failure, were prevented using the hybrid technology. There were no major complications, such as nerve root compression, dural tears, or wound infection in our study. In general, the most advantage of LP-PLSIF is less invasive compared with the traditional PLSIF [25, 27].

Our study has several limitations. Firstly, the 12-month follow-up period is relatively short. Secondly, the sample size was relatively small. Lastly, it is a retrospective cohort study and the case selection bias needs to be noticed. Although the fusion rate was higher in the LP-PLSIF group, we can’t draw a conclusion that LP-PLSIF can improve the fusion rate yet.

## Conclusion

LP-PLSIF is a feasible and effective surgical technique for lumbosacral degenerative diseases, especially for elderly patients with low bone mineral density. Compared with the traditional procedure, LP-PLSIF is less invasive and will accelerate the postoperative recovery of patients.

## Data Availability

Generated Statement: The raw data supporting the conclusions of this article will be made available by the authors, without undue reservation. If anyone needs the data and information from this study, please contact the first author of this paper.

## Abbreviations

ASD: Adjacent segment degeneration
BMD: Bone-mineral density
CT: Computed Tomograph
CBTS: Cortical bone trajectory screw
LP-PLSIF: Low-profile posterior lumbosacral interbody fusion
PLSIF: Posterior lumbosacral interbody fusion
PS: Pedicle screw
SAS: Sacral alar screw
MRI: Magnetic Resonance Imaging
VAS: Visual analogue scale
ODI: Oswestry Disability Index
JOA: Japanese Orthopaedic Association
